# Dissolved Heavy Metal Pollution and Assessment of a Karst Basin around a Mine, Southwest China

**DOI:** 10.3390/ijerph192114293

**Published:** 2022-11-01

**Authors:** Hong-Wei Liao, Zhong-Cheng Jiang, Hong Zhou, Xiao-Qun Qin, Qi-Bo Huang, Liang Zhong, Zheng-Gong Pu

**Affiliations:** 1 Key Laboratory of Geological Survey and Evaluation of Ministry of Education, China University of Geosciences, Wuhan 430074, China; 2 Institute of Karst Geology, Chinese Academy of Geological Sciences, Guilin 541004, China; 3 National Center for International Research on Karst Dynamic System and Global Change, Guilin 541004, China; 4 International Research Centre on Karst under the Auspices of United Nations Educational, Scientific and Cultural Organization, Guilin 541004, China

**Keywords:** karst water, dissolve heavy metals, health risk assessment, Pb–Zn mine, southwest China

## Abstract

Karst water quality is one of the most important environmental issues in karst areas. The study’s purpose was to investigate dissolved heavy metal pollution and health risk assessment in karst water basins around mines. River water and groundwater samples were analyzed by principal component analysis, correlation analysis, water quality index, hazard quotient, and hazard index. Median concentrations of dissolved heavy metals in the Sidi River were similar to the world average with a slightly alkaline characteristic. The concentrations of most dissolved heavy metals in river water were higher than those in groundwater. The concentrations of Zn, Pb, and Cd around the mine exceeded the limits of drinking water indicators. The poor water quality samples with high water quality index values were distributed around the mine. Lead (Pb), Zn, As, Cd, and Cr were potentially threatening metals in the study area. The pollution level of dissolved heavy metals in the Sidi River was at a medium level compared with other rivers worldwide. Principal component analysis and correlation analysis showed that Cu, Pb, Zn, Cd, Mn, Fe, As, and Sr mainly came from mine drainage; Ca^2+^, Mg^2+^, and Cr mainly came from the contribution of carbonate rocks; Na^+^ and K^+^ were related to local human agricultural activities. The concentrations of dissolved heavy metals in groundwater were affected by karst aquifers. The results of this study can provide a data reference for water resources prevention and human health protection in the Sidi River’s karst basin and similar karst basins.

## 1. Introduction

Water resources and water quality are water security problems facing the world [1]. In the process of the rapid development of the global economy, countries all over the world are facing the great challenge of water pollution [2], especially heavy metal pollution [3,4]. Heavy metals are toxic, persistent, and bio-accumulative [5,6]. Heavy metal elements in water are serious threats to human health and the ecosystem [7,8]. The sources of dissolved heavy metals mainly come from natural processes and human activities [9]. Natural processes include atmospheric precipitation, rock weathering, and volcanism [10]. Human activities include mining, metal smelting, industrial manufacturing, municipal sewage, and medical residues [11].

Karst aquifers are the source of drinking water for 20–25% of the world’s population [12,13]. Groundwater flows in karst aquifers have the characteristics of fracture flow and diffusion flow through a dual porous medium [14]. Karst conduits and fissures can result in strong interactions between surface water and groundwater, changing the chemical compositions of water [15]. These unique properties of karst aquifers make karst water extremely vulnerable to pollution caused by human activities, and it is difficult and time-consuming to repair. In the current situation, the pollution of dissolved heavy metals in karst water is very serious [16] and increasing [17]. Although many articles have reported the pollution and health risks of dissolved heavy metals in rivers [18,19,20], these paid more attention to the sources of heavy metals and their effects on water quality. However, the effects of specific water environments on the distribution characteristics of heavy metals are rarely considered [21]. The unique characteristics of karst aquifers may change the precipitation of dissolved heavy metals in karst water or the dissolution of particles containing heavy metals [14,15], which will affect the pollution level of heavy metals in water. However, study on the combination of karst aquifer environments and dissolved heavy metal pollution has not been reported. Karst water is one of the most important water resources to all of mankind [12]. The study on the pollution of heavy metals in karst water is beneficial for promoting the treatment and protection of water resources in karst areas around the world. Therefore, a study on the sources, distribution characteristics, and pollution level of dissolved heavy metals in karst basins is of great value.

The Sidi River’s karst basin was selected as the study area. The study area is an important supply source of the Lijiang River [22]. It plays an important supporting role in ensuring the safety of drinking water for urban and rural residents, the balance of the ecological environment, and rapid economic development. The Sidi River’s karst basin is located downstream of the Laochang Pb–Zn mine. Since the 1950s, the study area has been endangered by mine tailings and wastewater. Because of this incident, scholars have conducted a lot of research on the distribution characteristics, pollution degree, and restoration treatment of metals in the soil in the study area [22,23]. However, little attention has been paid to the pollution harm of dissolved heavy metals in the water of the study area. Therefore, several key points need to be addressed: (1) to what extent the river water and groundwater in the basin were polluted by dissolved heavy metals; (2) where the dissolved heavy metals came from; (3) whether the karst water basin influenced the spatial distribution of dissolved heavy metals and what the influencing factors were. To solve these problems, we systematically sampled the river water and groundwater in the Sidi River’s karst basin and tested the main physical and chemical indexes and the concentrations of dissolved metals in the samples. Principal component analysis, correlation analysis, water quality index, hazard quotient, and hazard index were analyzed systematically.

The objectives of this study were: (1) to identify the pollution sources of dissolved heavy metals in the study area; (2) to evaluate the effects of water quality and dissolved heavy metals on human health in the Sidi River’s karst water basin; and (3) to clarify the influencing factors of karst aquifer environments on the spatial distribution characteristics of dissolved heavy metals pollution in the study. It provided basic data for the water quality treatment, water resources protection, and human health protection of dissolved heavy metals in karst basins.

## 2. Materials and Methods

### 2.1. Study Area

**Figure 1:**
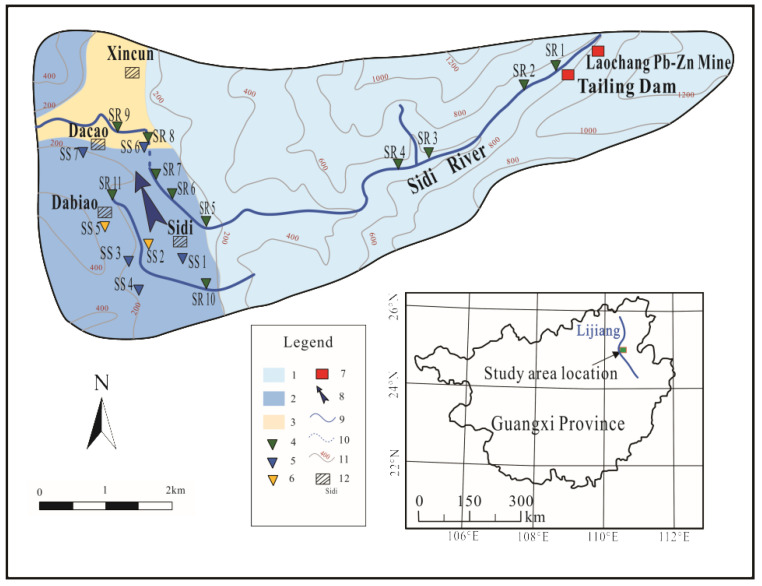
Hydrogeological sketch and sampling points distribution map of the study area. (1) Clastic rocks and carbonate with clastic rocks. (2) Carbonate rocks. (3) Carbonate rocks overlaid by Quaternary sediments. (4) Sampled surface water in river. (5) Sampled groundwater in springs. (6) Sampled groundwater in wells. (7) Tailing dump/mine. (8) Groundwater flowing direction. (9) River. (10) Cave river. (11) Contours and elevation. (12) Villages.

The Sidi River’s karst basin is located in the Guilin-Yangshuo basin in southwest China, covering an area of about 10 km^2^. The region is a subtropical monsoon climate, with 70 percent of the rainfall (1200 mm) occurring between April and August and an annual average temperature of 19 °C. The basin includes unconfined non-karstic aquifers of Cambrian–Devonian (Є-D) sandstones in the mountainous area to the east, unconfined Devonian (D) to Carboniferous (C) karst aquifers to the west, unconfined karst aquifers in Devonian (D) carbonates underlying discontinuity, Quaternary (Q) clay, and clay loam [24]. Karst aquifers provide drinking water for residents. A karst conduit and a surface river (the Sidi River) originate in the Sidi River’s karst basin. The Sidi River originates from the eastern mountain area where the Laochang Pb–Zn mine is located. The river water flows westward through Sidi village, northward through the karst conduit, and finally into the Dayuan River. The karst conduit is about 2 m high and 1 km long (Figure 1).

The Laochang Pb–Zn mine has been mined since the 1950s in the upper reaches of the Sidi River. The explored Pb–Zn ore dominantly contains sphalerite (ZnS) with Zn/Pb > 2, galena-sphalerite (PbS-ZnS) with Pb > Zn, and pyrite [25]. The mine wastewater resulted in serious pollution of the soil in the study area [26]. Mining activities were completely abandoned until 2012. A long mining history has resulted in the accumulation of heavy metals in the soil around Sidi village [24]. The average levels of zinc (Zn), lead (Pb), and copper (Cu) were 1442 mg/kg, 923 mg/kg, and 117 mg/kg in soil samples obtained in 2015, respectively. In addition, the content of cadmium (Cd) in the soil reached 40 mg/kg [22]. In February 2011, a local villager developed symptoms of cadmium poisoning [27]. Heavy metal pollution was serious in the study area.

### 2.2. Sampling and Analysis

The selection of sampling sites was based on the hydrological characteristics of the Sidi River and the type of groundwater. We sampled according to the flow direction of surface water and groundwater. Sampling was performed from 12 to 15 January 2022, including 11 river samples, 5 spring samples, and 2 well samples. We referred to spring water samples and well water samples collectively as groundwater samples. The sample locations are shown in Figure 1.

Water samples were collected from river water and groundwater. The pH and total dissolved solids (TDS) of water samples were immediately measured in the field by a portable multi-parameter water quality meter (WTW Multi 3430, Munich, Germany), with analytical uncertainties of 0.01 and 0.01 mg/L, respectively. The HCO_3_^−^ and Ca^2+^ concentrations were titrated in situ using the Merck titration box (Merck, NJ, USA), with analytical uncertainties of 0.1 mmol/L and 0.1 mg/L respectively. Samples for water chemistry analysis were infiltrated through a 0.45 μm filter immediately in situ and collected in three 550 mL polyethylene bottles. Polyethylene bottles were overflowed and tightly capped to protect from air contact and stored at 4 °C in a fridge after being sealed with parafilm. Water samples for the determination of cations and heavy metals were acidified with ultra-purified HNO_3_ (pH < 2). All samples were immediately transported to the laboratory for further analysis. Major cations in water samples were detected by full spectrum direct reading plasma spectrometer (IRIS Intrepid II XSP, Thermo Electron, Waltham, MA, USA). Anions in water samples were detected by ion chromatograph (ICS-2100, Dionex, Sunnyvale, CA, USA). The charge balance errors in all analyses were less than 8%. The detection limits for IRIS Intrepid II XSP and ICS-2100 were both 1 mg/L. Analytical precision for major ions was within 1%. Dissolved heavy metals in water samples were detected by inductively coupled plasma mass spectrometer (ICP-MS, Elan DRCE, PerkinElmer, Waltham, MA, USA). Quality assurance and quality control were assessed by standard operating procedures, calibration with standards, and analysis of reagent blanks, with each batch of 10 water samples. Relative standard deviations for dissolved heavy metals were ±5%, and the recovery percentage ranged from 90% to 110%. Otherwise, the samples were detected again until the data reached the standard.

### 2.3. Statistical Analysis

Multivariate statistical analysis is often used to interpret research data. The sources of dissolved heavy metals can be determined by principal component analysis (PCA). Correlations between heavy metals may provide information on the sources and migration of these elements [21]. The principal component analysis is to explore the sources of heavy metals by reducing the dimension of the data set to several influencing factors. Principal components (PC) with eigenvalues greater than 1 are retained [28]. The applicability of the data set to principal component analysis can be evaluated by the Kaiser–Meyer–Olkin (KMO) and Bartlett sphericity test (*p* < 0.001) [29]. Data analysis was carried out using SPSS 21.0 for Windows.

### 2.4. Water Quality Index

The water quality index (WQI) was used to obtain a comprehensive picture of river water quality [30]. Drinking water quality is calculated in Equation (1) [9,31]:

WQI = ∑[*W_i_* × (*C_i_/S_i_*) × 100] (1)

where *W_i_* is the weight of each element and represents different contributions to the overall water quality, which is calculated by the eigenvalues for each principal component (PC) and factor loading for each heavy metal from the PCA results. *C_i_* is the concentration of each heavy metal tested. *S_i_* represents the limit value of drinking water for each heavy metal. According to the WQI values, water quality can be classified into five categories as excellent water (WQI < 50), good water (50 ≤ WQI < 100), poor water (100 ≤ WQI < 200), very poor water (200 ≤ WQI < 300), and undrinkable water (WQI ≥ 300) [9,31].

### 2.5. Health Risk Assessment

Hazard index (HI) and hazard quotient (HQ) are usually considered in the studies of health risk assessment of metal elements in a water environment [32]. The hazard index (HI) is the sum of the two pathways of HQ and represents the total potential non-carcinogenic risk of each metal. If HQ or HI is more than 1, it indicates a potentially adverse effect on human health, and further research is needed [18]. The calculation method of the HQ and HI is calculated in Equations (2)–(6) [33]:

ADD_ingestion_ = (C*_w_* × IR × EF × ED)/(BW × AT) (2)

ADD_dermal_ = (C*_w_* × SA × K*_p_* × ET × EF × ED × 10^−3^)/(BW × AT) (3)

HQ = ADD/RfD (4)

RfD_dermal_ = RfD × ABS_GI_ (5)

HI = ∑HQs (6)

where ADD_ingestion_ and ADD_dermal_ are the average daily doses via ingestion or dermal exposure (mg/kg/day), respectively [33]. C_w_ is the heavy metal concentration of each sample (mg/L); IR is the ingestion rate (L/day); EF is the exposure frequency (day/year); ED is the exposure duration (years); BW is the body weight (kg); AT is the average time for non-carcinogens (days); SA is the area of exposed skin (cm^2^); Kp is the dermal permeability coefficient for each heavy metal in water (cm/h); ET is the exposure time (h/day); ABS_GI_ is the gastrointestinal absorption factor. The above parameters are from the United States Environmental Protection Agency (EPA) [32].

## 3. Results

The drinking water guidance values established by the China Environmental Protection Administration (2006) [34], U.S. Environmental Protection Agency (2004) [32], and World Health Organization (WHO) (2006) [35] were compared. The index value established by the China Environmental Protection Administration (2006) was used as the standard for evaluating water quality in the study. Kolmogorov–Smirnov (K-S) statistical data in the study area were used to test the normal distribution of data. The results showed that pH, K^+^, Na^+^, Mg^2+^, Cl^−^, NO_3_^−^, Cu, As, and Cr were in a normal distribution. However, The K-S results of the remaining elements had a large standard deviation (Table 1), indicating that their average concentrations may be affected by outliers [36]. Therefore, we used the median concentrations for analysis.

**Figure 2:**
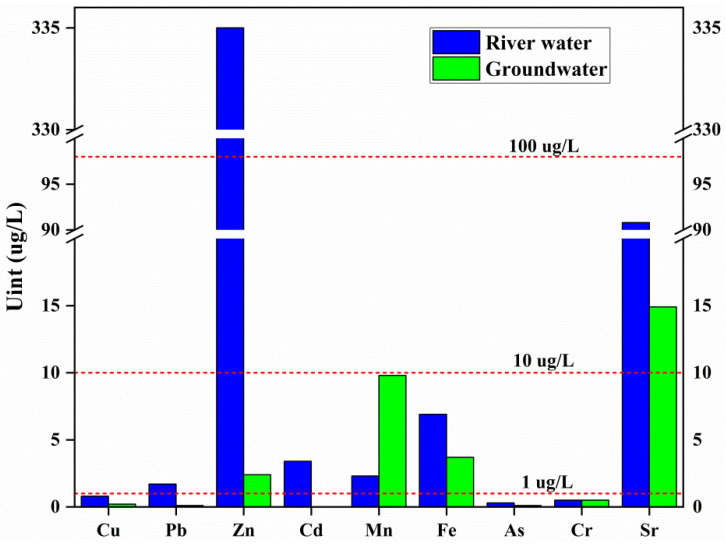
The median concentrations of dissolved heavy metals in the Sidi River’s basin.

The water was slightly alkaline in the study, and the median pH of the Sidi River’s water and groundwater were 7.4 and 7.5, respectively. The concentrations of TDS, Ca^2+^, Mg^2+^, and HCO_3_^−^ in groundwater were higher than those in river water. However, the concentrations of SO_4_^2−^ and Sr in river water were higher than those in groundwater (Table 1). The median concentrations of dissolved heavy metals in the study were all within the limits of drinking water indicators (Table 1, Figure 2), indicating that the pollution of dissolved heavy metals in the study was not serious. However, the concentrations of some dissolved heavy metals exceeded the limits at specific locations (Zn, Pb, and Cd in SR1 and SR2, Pb in SR3). The positions of the samples of dissolved heavy metals were close to the tailing dam. According to the median concentrations of dissolved heavy metals, metal elements were divided into three categories. Zinc (>100 μg/L) was the most abundant element; Sr, Mn, Fe, Cd, and Pb (1 to 10 μg/L) were moderately rich elements; and Cu, As, and Cr (<1 μg/L) belonged to low-abundance elements (Figure 2). The concentrations of dissolved heavy metals in river water were higher than those in groundwater (except for Cr), showing that the river was more affected by human activities than groundwater.

## 4. Discussion

### 4.1. Principal Component Analysis

The correlation analysis of dissolved heavy metals in the study area is shown in Table 2. The significant positive correlation between Na^+^ and K^+^ (0.913) indicated that the sources of the two elements were similar. There were significant positive correlations (>0.79) among Cu, Pb, Zn, Cd, Mn, Fe, As, and Sr, indicating that the sources of these elements were similar. There were significant positive correlations (>0.64) among Ca^2+^, Mg^2+^, and Cr, indicating that the three elements had similar sources [18,21].

**Figure 3:**
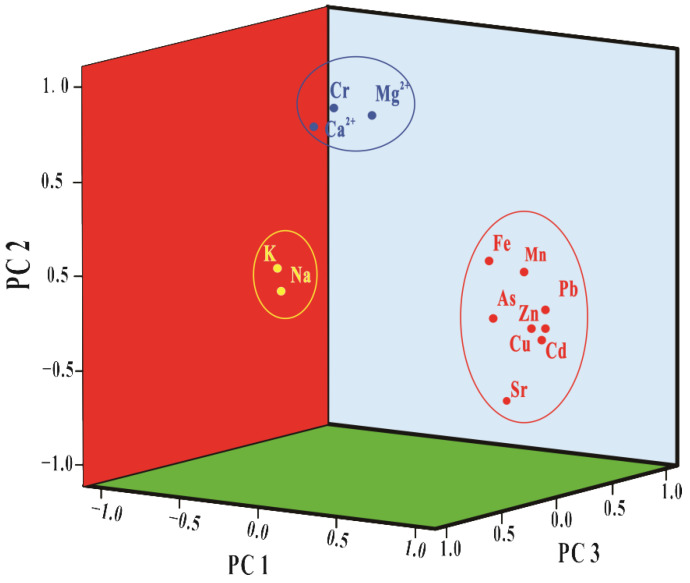
Principal component analysis for dissolved metals in the Sidi River’s karst basin.

Principal component analysis (PCA) was used to identify the sources of dissolved metals in the study area [37,38]. The Kaiser–Meyer–Olkin and Bartlett values of the test results (0.732 and 0.000, respectively) showed that the data of this study were suitable for principal component factor loading analysis [29]. Three principal components (PC1, PC2, and PC3) were extracted from 13 elements in the Sidi River’s karst basin. The sum of the variance of these three principal components was 90.55% (Table 3, Figure 3).

A total of 55.42% of the variance was explained by Cu, Pb, Zn, Cd, Mn, Fe, As, and Sr. The median concentrations of dissolved Pb, Zn, and Cd in the study area (1.7 μg/L, 335 μg/L, and 3.4 μg/L, respectively) were much larger than the background concentrations of the Lijiang River (0.05 μg/L, 14.81 μg/L, and 0.02 μg/L, respectively) [19], indicating that the natural source was not the main input end member of these dissolved metals. The study area is located downstream of the Laochang Pb–Zn mine, which can provide a source of dissolved heavy metals. Lead–zinc mines are rich in polymetallic elements (e.g., Pb, Zn, and Cd) [39,40,41]. Lead–zinc mines usually also contain small amounts of iron sulfide and copper sulfide [42,43]. In the action of leaching and mine drainage, metal elements in tailings sediments and wastewater were released into the Sidi River. Therefore, principal component 1 (PC1) came from the contribution of the Laochang Pb–Zn mine. The very high concentrations of dissolved heavy metals in the river water near the tailing dam and significant correlations among these elements also supported this view (Table 2). Principal component 2 (PC2) caused 21.41% of the variance, with high loading values of Ca^2+^, Mg^2+^, and Cr. The strata in the study area mainly include limestone, dolomite, and dolomitic limestone. The dissolution of carbonate will increase the concentrations of Ca^2+^ and Mg^2+^ in water [24]. The concentrations of Ca^2+^ and Mg^2+^ in the downstream karst area (69.2 mg/L, 15.3 mg/L) were significantly higher than those in the upstream non-karst area (29.9 mg/L, 8.0 mg/L), indicating that the dissolved Ca^2+^ and Mg^2+^ mainly came from the process of weathering and the dissolution of carbonate. There were also significant correlations among Ca^2+^, Mg^2+^, and Cr (Table 2). Therefore, PC2 came from the input of carbonate strata. Principal component 3 caused 14.72% of the variance, with high loading values of Na^+^ and K^2+^. The study area was far away from the ocean, so sea salts were not the main sources of Na^+^ and K^+^ in the Sidi River’s karst basin [44,45]. The median value of (Na^+^+K^+^)/Cl^−^ in the study area was 1.3 (>1), indicating that Na^+^ and K^+^ not only came from evaporite (KCl and NaCl) [46] but also from the inputs of other contributors. Nitrate is usually used as a characteristic ion reflecting human activities [46]. The maximum concentration of NO_3_^−^ in the study area (37.4 mg/L) was much higher than the local water’s background value (4.9 mg/L) [47], indicating that the study area was significantly affected by human activities. Therefore, the concentrations of Na^+^ and K^+^ were mainly from the uses of different types of potassium and sodium fertilizers (e.g., manure and urea) and pesticides in local human agricultural activities. The high concentrations of Na^+^ and K^+^ near farmlands and villages also indicated that PC3 came from human activities.

### 4.2. Spatial Distribution Characteristics of Dissolved Heavy Metals

**Figure 4:**
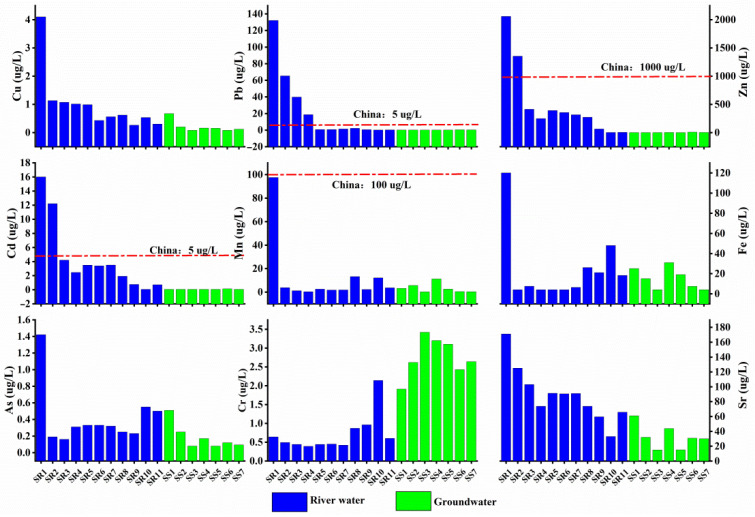
Distribution characteristics of heavy metals in river water and groundwater.

Compared with the river water, the median concentrations of dissolved heavy metals in the groundwater (except for Cr) decreased (Figure 4). Karst aquifers can react with mine wastewater to form metal complex precipitations [48], resulting in a decrease in the concentrations of dissolved heavy metals in the groundwater. Bacteria sulfate reduction (BSR) can also form highly insoluble metal sulfide deposits in karst areas [49]. In the process of entering karst water from river water, the concentration of SO_4_^2−^ (from 34.6 mg/L to 11.4 mg/L) decreased obviously, indicating the existence of BSR. In the process of river seepage to karst aquifers, some heavy metal particles will be trapped in karst fissures, which also reduced the concentrations of dissolved heavy metals in groundwater [50]. Moreover, the flow speed of groundwater in the Sidi River’s basin was slower, which was more conducive to the formation of heavy metal precipitation. The increase in Cr concentration was from the dissolution of limestone, and principal component analysis also indicated that Cr mainly comes from strata.

Compared with the river sample SR7 at the entrance of the conduit, the concentration of Pb, Mn, and Fe from the SR8 at the outlet of the conduit increased slightly (varying from 1.3 μg/L to 2.1 mg/L, from 1.8 μg/L to 13.1 mg/L, and from 6.3 μg/L to 20.6 mg/L, respectively) (Figure 4). The reason may be that large proportions of heavy metals (Pb, Mn, and Fe) in the particles were re-dissolved in the karst groundwater in the action of mixed water. This phenomenon also was observed in a Pb–Zn mine [51] and mine wastewater in northeastern Italy’s Alps [52]. The increased Fe and Mn concentrations may be from the dissolution of carbonate minerals. In the karst conduit, the mixed water containing mine wastewater further eroded the karst aquifer’s rocks. Moreover, the similar concentration of Fe and Mn in groundwater and conduit water also proved this view. Arsenic adsorbed in particulates can also be desorbed into water under aerobic and anaerobic conditions [53,54]. As a result, the concentration of dissolved arsenic in karst water increased (SR10, SR11, and SS1) (Figure 4). This phenomenon also occurred in another karst area: southwest China [55].

### 4.3. Dissolved Heavy Metals in Sidi River and Other Rivers

The comparisons of dissolved heavy metal contents are shown in Table 4. Except for the heavy metal elements (Pb, Zn, and Cd) seriously affected by the mine, the concentrations of other dissolved heavy metals in the Sidi River were similar to the world average [56]. The concentrations of dissolved heavy metals in the Sidi River were also similar to those in the upper reaches (the Xijiang River) [20], the lower reaches (the Pearl River) [57], and the background value of the study area (the Lijiang River) [19]. It may be that these river basins were mainly carbonates, and the contributions of rocks to heavy metals were similar. The concentrations of dissolved heavy metals in the Sidi River were significantly lower than those in the Huanghe River [58], the Huaihe River [18], and the Changjiang River [59], which may be that the contributions of frequent human activities were main sources of heavy metals in these rivers. Compared with the rivers of other countries, the concentrations of dissolved heavy metals in the Sidi River were higher than those in some developed countries [60,61] but lower than those in some developing countries [62,63]. This may be related to the level of prevention and management of dissolved heavy metals in different countries. Therefore, the heavy metal pollution level in the Sidi River was at a medium level.

### 4.4. Water Quality Index and Health Risk Assessment

**Figure 5:**
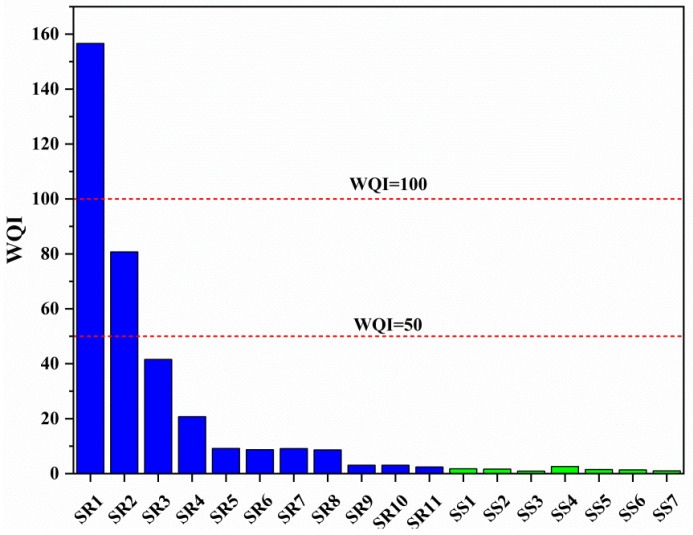
The water quality index (WQI) values of water in the Sidi River’s karst basin.

The water quality index (WQI) value of each sampling point is calculated in Equation (1) (Figure 5). The WQI values in the Sidi River’s karst basin varied from 0.9 to 156.6, with an average of 19.7. Sample SR1 (WQI = 156.6) was poor water; sample SR2 (WQI = 80.7) was good water; and other water samples (WQI < 50) were excellent water. Eighty-two percent of the samples in the Sidi River were excellent water, and all the samples in the groundwater were excellent water. The poor water was because the sampling locations (SR1 and SR2) were closer to the tailings dam and more seriously affected by mine drainage (Figure 1).

According to PCA, we calculated the weight of each dissolved heavy metal in the water of the study area (Table 5). The calculated method of the HQ and HI was shown in Equations (2)–(6). The HQ_ingestion_, HQ_dermal_, and HI values were much less than 1 (Table 6), indicating that these elements were less harmful. Compared with dermal, ingestion was the main route, by which these dissolved heavy metals harmed humans. Children’s HQ_ingestion_, HQ_dermal_, and HI values were higher than adults, indicating that children were more vulnerable than adults exposed to the same environment. The H_Qingestion_ and HI of As and the HQ_dermal_ of Cd were highest in river water, and the H_Qingestion_ and HI of As and the HQ_dermal_ of Cr were highest in groundwater, indicating their potential hazards.

The results showed that As was the most potentially threatening metal in the Sidi River’s karst basin, and long-term consumption of As may result in underlying diseases [64]. Residents should attach great importance to As in water, and it is necessary to take scientific measures to remove As in natural water. In addition, As, Pb, Zn, Cd, and Cr cannot be ignored either. Moreover, heavy metal particles deposited in river water will re-dissolve into the river water [65], posing a potential threat to the river water. Impacted by mining, there were a large number of heavy metal particles in the soil and rock fissures in karst areas [23]. In the process of seepage caused by rainfall, these particles will migrate again [66], threatening local water quality. Residents should strengthen the corresponding preventions and controls.

## 5. Conclusions

As the source of drinking water for 20–25% of the world’s population, karst water quality is one of the most important environmental issues in the sustainable development of karst areas. We studied the geochemical characteristics of major ions and dissolved heavy metals in the Sidi River’s water and groundwater. The results showed that Cu, Pb, Zn, Cd, Mn, Fe, As, and Sr in the Sidi River’s karst basin mainly came from the discharge of mine wastewater (55.42% of the variance); Ca^2+^, Mg^2+^, and Cr mainly came from the contribution of weathering and dissolution of carbonate rocks (21.41% of the variance); Na^+^ and K^+^ were related to local human agricultural activities (14.72% of the variance). The concentrations of some dissolved heavy metals exceeded the limits at specific locations (Zn, Pb, and Cd in SR1 and SR2, Pb in SR3). The concentrations of dissolved heavy metals in river water (except for Cr) were higher than that in groundwater. In the effect of dilution and deposition, the concentrations of dissolved heavy metals in river water decreased, as these were farther away from the tailing reservoir. The concentration of Cr from carbonate rocks in groundwater was higher than that in river water. The concentrations of heavy metals in groundwater were significantly reduced under the combined action of the porous media properties of carbonate, the buffering effect, and the bacteria sulfate reduction (BSR). In the action of mixed water, a large proportion of heavy metals in the particles (e.g., Pb, Mn, and Fe) and As adsorbed on the particles was re-dissolved in water, resulting in these concentrations increasing slightly. Compared with other rivers in the world, the pollution level of dissolved heavy metals in the Sidi River was in the middle level. Stratigraphic lithology, human activities, and the level of prevention and management of heavy metal pollution may have an important impact on the dissolved heavy metals in rivers. Except for samples SR1 and SR2, the water quality indexes of other samples were less than 50 in the Sidi River. For health risk assessment, all HQingestion, HQdermal, and HI values were below one. Lead (Pb), Zn, As, Cd, and Cr were potentially threatening metals in the study area. Moreover, heavy metal particles in water, soil, and rock fissures may dissolve and re-migrate, potentially threatening the local water quality and health of residents. Residents should strengthen the pollution control of dissolved heavy metals. The results of this study can provide a data reference for water resources prevention and human health protection in the Sidi River’s karst basin and similar karst basins.

## Figures and Tables

**Table 1:** Statistics of physicochemical and chemical parameters and the parameters for water quality index (WQI) calculation in river water and groundwater in the Sidi River’s karst basin.

Parameters	Surface Water					Ground Water					China [34]
	Max	Min	Mean	Median	SD	Max	Min	Mean	Median	SD	
pH	7.6	7.1	7.4	7.4	0.2	8.1	7.3	7.58	7.5	0.2	6.5~8.5
TDS (mg/L)	204.9	73.2	123.5	115.6	41.4	263.8	193.6	227	223.6	21.8	1000
K^+^ (mg/L)	2.9	0.3	0.8	0.6	0.7	5	0.1	1.6	1.1	1.8	
Na^+^ (mg/L)	3	1.2	1.7	1.6	0.5	5.7	0.2	2.3	1.6	2	200
Ca^2+^ (mg/L)	69.9	22.2	31.3	29.9	13.8	98	61.9	75.9	69.2	14	
Mg^2+^ (mg/L)	14.8	5.2	9	8	3.3	29.6	5.6	15.9	15.3	8.1	
SO_4_^2−^ (mg/L)	142.2	12.8	44.1	34.6	36.9	16.3	4.9	11.1	11.4	3.2	250
HCO_3_^−^ (mg/L)	242.5	56.6	90.6	66.7	54	347.6	231.4	282.5	282.9	33.2	
Cl^−^ (mg/L)	6.6	0.8	1.6	1	1.7	12.3	1	4.7	2.8	3.8	250
NO_3_^−^ (mg/L)	13.4	1.9	6.5	6.7	3.2	37.4	3.5	15.1	13.5	10.2	20
Cu (μg/L)	4.1	0.3	1.1	0.8	1	0.7	0.1	0.2	0.2	0.2	1000
Pb (μg/L)	132	0.1	26.1	1.7	41	0.4	0.1	0.2	0.1	0.1	10
Zn (μg/L)	2057	2.1	547	335	613.1	7.5	0.7	2.3	1.1	2.4	1000
Cd (μg/L)	16	0.1	4.8	3.4	4.9	0.1	0.1	0.1	0.1	0	5
Mn (μg/L)	97.5	0.3	13.6	2.3	28.3	11.1	0.2	3.3	2.5	9.8	100
Fe (μg/L)	120	4	24.5	6.9	34.6	31	4	15	15	3.7	300
As (μg/L)	1.4	0.2	0.4	0.3	0.4	0.5	0.1	0.2	0.1	0.1	10
Cr (μg/L)	2.1	0.4	0.7	0.5	0.5	3.4	1.9	2.8	2.6	0.5	50
Sr (μg/L)	171	33	91.2	90.8	35.6	60.9	14.9	32.5	30.7	14.9	

**Table 2:** The correlation analysis of dissolved heavy metals in the study area.

	K^+^	Na^+^	Ca^2+^	Mg^2+^	Cu	Pb	Zn	Cd	Mn	Fe	As	Cr	Sr
K^+^	1												
Na^+^	0.913 **	1											
Ca^2+^	0.427	0.350	1										
Mg^2+^	−0.142	−0.236	0.342	1									
Cu	−0.056	−0.034	−0.405	−0.082	1								
Pb	−0.164	−0.111	−0.331	0.053	0.930 **	1							
Zn	−0.221	−0.149	−0.450	−0.009	0.905 **	0.954 **	1						
Cd	−0.246	−0.163	−0.496*	−0.038	0.874 **	0.939 **	0.995 **	1					
Mn	0.033	0.046	−0.117	0.095	0.904 **	0.831 **	0.779 **	0.719 **	1				
Fe	0.247	0.222	0.018	0.102	0.792 **	0.698 **	0.613 **	0.546 *	0.948 **	1			
As	0.212	0.152	−0.261	−0.106	0.868 **	0.714 **	0.683 **	0.639 **	0.895 **	0.890 **	1		
Cr	0.273	0.195	0.917 **	0.641 **	−0.459	−0.375	−0.492 *	−0.539 *	−0.162	−0.042	−0.354	1	
Sr	−0.153	−0.043	−0.486 *	−0.387	0.835 **	0.807 **	0.884 **	0.901 **	0.614 **	0.454	0.648 **	−0.442 *	1

Notes: **, the correlation is significant at a confidence level (one test) of 0.01; *, the correlation is significant at a confidence level (one test) of 0.05. Bold text indicates significant correlations.

**Table 3:** Pearson correlation matrix of heavy metals in the Sidi River’s karst basin.

Parameter	PC1	PC2	PC3	Communalities
K^+^	0.01	0.12	**0.95**	0.92
Na^+^	0.02	0.01	**0.94**	0.89
Ca^2+^	−0.21	**0.80**	0.39	0.84
Mg^2+^	0.12	**0.81**	−0.35	0.80
Cu	**0.95**	−0.24	−0.04	0.96
Pb	**0.93**	−0.14	−0.18	0.92
Zn	**0.89**	−0.27	−0.23	0.93
Cd	**0.85**	−0.33	−0.26	0.90
Mn	**0.96**	0.08	0.07	0.94
Fe	**0.89**	0.17	0.28	0.89
As	**0.88**	−0.15	0.24	0.85
Cr	−0.25	**0.94**	0.18	0.98
Sr	**0.72**	−0.46	−0.10	0.96
Eigenvalues (%)	7.08	2.78	1.91	
Variance (%)	55.42	21.41	14.72	
Cumulative (%)	55.42	75.83	90.55	

Notes: Extraction method: principal component analysis. Factor loadings beyond −0.6 to 0.6 are marked by bold font.

**Table 4:** Comparison of the concentrations of dissolved heavy metals (μg/L) in the Sidi River’s water with other rivers in the world.

Rivers	Cu	Pb	Zn	Cd	Mn	Fe	As	Cr	Sr	References
Sidi River, China	0.8	1.7	335.0	3.4	2.3	6.9	0.3	0.5	90.8	This study
Lijiang River, China	0.66	0.05	14.81	0.02	23.96	—	1.13	1.62	—	[19]
Xijiang River, China	1.01	0.1	1.82	0.01	0.30	—	—	0.33	259	[20]
Pearl River, China	1.09	0.08	3.61	0.04	1.06	—	—	1.70	—	[57]
Huanghe River, China	4.2	3.9	24.8	0.05	—	—	1.9	—	—	[58]
Huai River, China	52.3	155	10504	61.7	49.0	441		23.1	—	[18]
Changjiang River, China	8.40	6.40	18.75	0.28	—	1660	7.00	8.90	—	[59]
Catalan River, Spain	1.3	2.2	1.9	1.2	—	—	2.9	2.4	—	[60]
Trinity River, USA	1.2	0.03	—	0.01	4.2	5.8	—	—	—	[61]
To Lich River, Vietnam	4.5	8.1	51.1	—	216	—	39.1	2.9	—	[62]
Damodar River, India	18	10	89	9	33	—	—	16	—	[63]
World average	1.48	0.08	0.60	0.08	34.0	66.0	0.62	0.70	60.0	[56]

Note: —, nonavailability of data.

**Table 5:** Hazard quotient and hazard index for each heavy metal in the Sidi River’s karst basin.

PC	Eigenvalues	Relative Eigenvalue	Parameter	Loading Value	Relative Loading Value on the Same PC	Weight
F1	7.08	0.60	Cu	0.95	0.13	0.08
			Pb	0.93	0.13	0.08
			Zn	0.89	0.13	0.08
			Cd	0.85	0.12	0.07
			Mn	0.96	0.14	0.08
			Fe	0.89	0.13	0.08
			As	0.88	0.12	0.07
			Sr	0.72	0.10	0.06
			Total	7.07	1.00	0.60
F2	2.78	0.24	Ca^2+^	0.8	0.31	0.07
			Mg^2+^	0.81	0.32	0.08
			Cr	0.94	0.37	0.09
			Total	2.55	1	0.24
F3	1.91	0.16	K^+^	0.95	0.50	0.08
			Na^+^	0.94	0.50	0.08
			Total	1.89	1.00	0.16
Total	11.77					1.00

Note: weight was calculated by relative eigenvalue times relative loading value.

**Table 6:** Reference dose (RfD), hazard quotient (HQ), and hazard index (HI) for each element in river water and groundwater in the Sidi River’s karst basin.

Element	K_p_ [32,67]	RfD_ingestion_ [21] (μg/kg/day)	RfD_dermal_ [21] (μg/kg/day)	HQ_ingestion_		HQ_dermal_		HI = ΣHQs	
				Adult	Child	Adult	Child	Adult	Child
River water									
Cu	1 × 10^−3^	40	12	3.36 × 10^−4^	3.49 × 10^−4^	1.03 × 10^−5^	2.11 × 10^−5^	3.46 × 10^−4^	3.70 × 10^−4^
Pb	1 × 10^−4^	1.4	0.42	4.23 × 10^−3^	4.40 × 10^−3^	6.31 × 10^−5^	1.29 × 10^−4^	4.30 × 10^−3^	4.53 × 10^−3^
Zn	6 × 10^−4^	300	60	6.57 × 10^−3^	6.87 × 10^−3^	5.15 × 10^−4^	1.06 × 10^−3^	7.08 × 10^−3^	7.93 × 10^−3^
Cd	1 × 10^−3^	0.5	0.025	9.96 × 10^−3^	1.04 × 10^−2^	2.08 × 10^−3^	4.28 × 10^−3^	1.20 × 10^−2^	1.46 × 10^−2^
Mn	1 × 10^−3^	24	0.96	1.72 × 10^−4^	1.79 × 10^−4^	3.75 × 10^−5^	7.69 × 10^−5^	2.09 × 10^−4^	2.56 × 10^−4^
Fe	1 × 10^−3^	700	140	4.07 × 10^−6^	4.24 × 10^−6^	7.60 × 10^−7^	1.57 × 10^−6^	4.83 × 10^−6^	5.81 × 10^−6^
As	3 × 10^−2^	0.3	0.285	2.80 × 10^−2^	2.91 × 10^−2^	1.62 × 10^−4^	3.33 × 10^−4^	2.82 × 10^−2^	2.94 × 10^−2^
Cr	1 × 10^−3^	3	0.075	1.87 × 10^−4^	1.94 × 10^−4^	3.08 × 10^−4^	6.34 × 10^−4^	4.95 × 10^−4^	8.27 × 10^−4^
Sr	1 × 10^−3^	600	120	4.15 × 10^−3^	6.19 × 10^−3^	1.08 × 10^−4^	3.18 × 10^−4^	4.26 × 10^−3^	6.51 × 10^−3^
Groundwater									
Cu	1 × 10^−3^	40	12	8.40 × 10^−5^	8.72 × 10^−5^	2.56 × 10^−6^	5.29 × 10^−6^	8.66 × 10^−5^	9.25 × 10^−5^
Pb	1 × 10^−4^	1.4	0.42	2.49 × 10^−4^	2.59 × 10^−4^	3.71 × 10^−6^	7.61 × 10^−6^	2.53 × 10^−4^	2.67 × 10^−4^
Zn	6 × 10^−4^	300	60	2.16 × 10^−5^	2.26 × 10^−5^	1.69 × 10^−6^	3.48 × 10^−6^	2.33 × 10^−5^	2.60 × 10^−5^
Cd	1 × 10^−3^	0.5	0.025	2.93 × 10^−4^	3.05 × 10^−4^	6.13 × 10^−5^	1.26 × 10^−4^	3.54 × 10^−4^	4.31 × 10^−4^
Mn	1 × 10^−3^	24	0.96	1.87 × 10^−4^	1.94 × 10^−4^	4.07 × 10^−5^	8.36 × 10^−5^	2.28 × 10^−4^	2.78 × 10^−4^
Fe	1 × 10^−3^	700	140	8.85 × 10^−6^	9.22 × 10^−6^	1.65 × 10^−6^	3.40 × 10^−6^	1.05 × 10^−5^	1.26 × 10^−5^
As	3 × 10^−2^	0.3	0.285	9.34 × 10^−3^	9.70 × 10^−3^	5.41 × 10^−5^	1.11 × 10^−4^	9.39 × 10^−3^	9.82 × 10^−3^
Cr	1 × 10^−3^	3	0.075	9.73 × 10^−4^	1.01 × 10^−3^	1.60 × 10^−3^	3.29 × 10^−3^	2.57 × 10^−3^	4.30 × 10^−3^
Sr	1 × 10^−3^	600	120	1.40 × 10^−3^	2.09 × 10^−3^	3.66 × 10^−5^	1.08 × 10^−4^	1.44 × 10^−3^	2.20 × 10^−3^
